# Associations Between Serum Iron Biomarkers and Breast Cancer Tumor Size

**DOI:** 10.1158/2767-9764.CRC-23-0205

**Published:** 2024-01-23

**Authors:** Ann Von Holle, Rachel L. Thompson, Katie M. O'Brien, Dale P. Sandler, Clarice R. Weinberg

**Affiliations:** 1 Biostatistics and Computational Biology Branch, National Institute of Environmental Health Sciences, Durham, North Carolina.; 2 Department of Environmental, Occupational, and Geospatial Health Sciences, City University of New York Graduate School of Public Health & Health Policy, New York, New York.; 3 Epidemiology Branch, National Institute of Environmental Health Sciences, Durham, North Carolina.

## Abstract

Excess iron has been shown to promote tumor growth in animals whereas iron deficiency has been associated with reduced or slowed tumor growth. The objective of this analysis was to estimate the associations between serum iron biomarkers and tumor size at diagnosis and metastatic status in a sample of breast cancer cases from the Sister Study.

The analytic sample included 2,494 incident breast cancer cases with information on tumor size and iron biomarkers, including serum iron (mcg/dL), ferritin (mcg/dL), and percent transferrin saturation, measured in serum collected at baseline. We used Spearman rank correlation and linear regression models to assess the associations between one SD changes in serum iron biomarker levels and natural log of tumor size (cm) adjusting for body mass index and age at study entry.

We did not find strong associations between any of the three serum iron biomarkers and tumor size. Adjusted regression slopes (95% confidence interval) were −0.016 (−0.048 to 0.016) for serum iron, −0.032 (−0.064 to <0.001) for ferritin, and −0.010 (−0.043 to 0.023) for transferrin saturation.

This study did not provide evidence supporting the hypothesis of a positive association between breast cancer tumor size at diagnosis and prediagnostic serum iron levels. Conflicting evidence between this study and previous research in animal models suggests that iron in the human tumor microenvironment may operate independently of circulating iron or body iron stores.

Iron has shown protumorigenic activity in animal models, but our data do not support a positive relationship between breast tumor growth and iron status.

**Significance:**

Using a large sample of women from a U.S. prospective cohort, we assessed associations between several serum iron measures at baseline and breast cancer tumor size and metastatic status. All estimated associations were close to zero with no evidence to support our hypothesis of higher body iron levels associated with larger tumor size. These results suggest the human tumor microenvironment operates independently of circulating serum iron levels.

## Introduction

Disruption of iron metabolism occurs in cancer cells (1), and iron is associated with aggressive tumor cell behavior (2). Excess iron can promote tumor growth in animal models (3). Inversely, low levels of iron are associated with reduced tumor growth, and iron chelation or iron depletion mechanisms are associated with better outcomes in animal models for metastasis (4, 5).

The association between iron levels and breast cancer incidence has been the subject of extensive research with inconsistent results across studies (6). However, few observational human studies have examined the link between tumor characteristics and iron levels. It is possible that mechanisms and their related factors associated with breast cancer incidence are independent of those promoting breast cancer tumor growth. Research in this area can help inform potential mechanisms for tumor growth in the presence of a disrupted iron metabolism or disrupted regulation of iron absorption or iron overload.

Given the deleterious effects of higher serum iron levels on tumor size in animal models, we hypothesized a similar direction of association when using observational data. Our aim in this study was to evaluate associations between three common serum iron measures and tumor size using a large U.S.-based prospective cohort study.

## Materials and Methods

We used cases from a case-cohort sample from the Sister Study, a cohort including 50,884 women who never had a breast cancer diagnosis at enrollment and had at least one sister diagnosed with breast cancer (7). Participants provided written informed consent and the study is overseen by the Institutional Review Board of the NIH in accordance with the U.S. Common Rule. Among the participants with baseline serum iron measures and follow up time before October 2017, data release 7.1 (ref. 8; *n* = 6,008), there were 2,494 women with a tumor size measure from the total of 3,007 diagnosed with invasive or ductal carcinoma *in situ* breast cancer (Supplementary Fig. S1).

Assays were based on a blood draw at baseline, and methods used for laboratory analysis have been described previously (7, 8). Serum iron levels included assays for levels of iron (mcg/dL), transferrin saturation (%), and ferritin (mcg/dL). Tumor size (cm), grade, stage, and metastatic status were extracted from medical records (7).

Descriptive statistics included median and interquartile ranges (IQR) for continuous variables and frequencies for categorical variables. We used linear regression models to estimate the association between one SD increase in serum iron levels and tumor size with and without adjustment for age at baseline and body mass index (BMI). Following regression diagnostics including visual examination of residuals, we used a natural log transform for the tumor size outcome. We used logistic regression models to estimate ORs of binary metastatic status at diagnosis (yes/no) for a one SD change in continuous serum iron level exposures. If a participant had multiple tumors, we included the largest tumor as the outcome.

We conducted several sensitivity analyses. First, we estimated the associations between serum iron levels and tumor size after partitioning the sample as follows: (i) for diagnoses occurring between 6 months and 4 years after study entry, (ii) excluding women who reported taking iron supplements, and (iii) stratifying by tumor subtype. We also estimated median tumor size within categories defined by extreme serum iron levels and menopause status at baseline. Finally, we evaluated the association between serum iron levels and breast cancer stage and grade using a nonparametric Kruskal–Wallis rank-sum test.

### Data Availability

Data described in the article and analytic code will be made available upon request (https://sisterstudy.niehs.nih.gov/).

## Results

The median (IQR) age of women in the analytic sample was 57 (51–64) years and most women were postmenopausal (70%) (Table 1) at recruitment. The median (IQR) largest tumor size was 1.4 cm (0.8–2.1) and median time from blood draw to diagnosis for this group was 4.6 years. Most women in the sample had one tumor (92%), and almost all tumors were non-metastatic (99%).

**TABLE 1:** Sample characteristics^a^

Characteristic	Cases with tumor data, *N* = 2,494	Cases missing tumor data, *N* = 513
Baseline age (years)	57 (51–64)	56 (50–64)
Age at menarche	13.00 (12.00–13.00)	12.50 (11.50–13.00)
Serum iron (mcg/dL)	94 (75–116)	91 (72–115)
Ferritin (mcg/dL)	68 (37–115)	78 (43–122)
Transferrin iron saturation (%)	29 (22–36)	28 (22–36)
Largest tumor (cm)	1.40 (0.80–2.10)	
Number of tumors		
1	2,285 (92%)	144 (95%)
2	191 (7.7%)	7 (4.6%)
3	18 (0.7%)	0 (0%)
Metastatic status (yes)	14 (0.6%)	7 (2.2%)
Follow-up time (years)	4.60 (2.40–7.00)	4.20 (2.10–6.90)
Postmenopausal status (yes)	1,758 (70%)	355 (69%)
Age at recruitment >50 years (yes)	1,912 (77%)	384 (75%)
Race/ethnicity		
Non-Hispanic White	2,182 (88%)	402 (78%)
Non-Hispanic Black	155 (6.2%)	68 (13%)
Hispanic	87 (3.5%)	28 (5.5%)
Other	69 (2.8%)	15 (2.9%)
BMI (kg/m^2^)	27 (24–31)	28 (24, 32)
BMI categories		
<18.5	22 (0.9%)	4 (0.8%)
18.5 to 24.9	894 (36%)	173 (34%)
25.0 to 29.9	817 (33%)	150 (29%)
30.0 and above	761 (31%)	186 (36%)
Stage		
0	469 (19%)	156 (51%)
I	1,360 (55%)	84 (27%)
II	520 (21%)	47 (15%)
III	96 (3.9%)	12 (3.9%)
IV	14 (0.6%)	7 (2.3%)
Grade		
1	558 (28%)	8 (19%)
2	918 (46%)	15 (35%)
3	503 (25%)	20 (47%)

^a^Median (IQR); n (%).

The nonparametric Spearman rank correlation statistics reflected near null findings (Table 2). Adjusted regression slopes (95% confidence interval) were −0.016 (−0.048 to 0.016) for serum iron, −0.032 (−0.064 to <0.001) for ferritin, and −0.010 (−0.043 to 0.023) for percent transferrin saturation (Table 2). ORs for the association between a metastatic cancer and iron levels were near 1, the adjusted ORs ranging from 1.12 for serum iron and 0.97 for transferrin saturation, with wide 95% confidence intervals.

**TABLE 2:** Associations between iron serum biomarkers, tumor size, and metastatic status

		Largest tumor size^a^		Metastatic status	
Exposure^b^	Spearman rank correlation	Unadjusted	Adjusted^c^	OR (95% CI)	OR (95% CI)^c^
Iron (mcg/dL)	−0.017	−0.016 (−0.048 to 0.015)	−0.016 (−0.048 to 0.016)	1.109 (0.659 to 1.868)	1.119 (0.650 to 1.926)
Ferritin (mcg/dL)	−0.031	−0.037 (−0.068 to −0.006)	−0.032 (−0.064 to 0.000)	1.138 (0.832 to 1.557)	1.137 (0.825 to 1.568)
Transferrin saturation (%)	−0.017	−0.010 (−0.042 to 0.022)	−0.010 (−0.043 to 0.023)	0.965 (0.556 to 1.678)	0.968 (0.550 to 1.705)

^a^Natural log-transformed.

^b^Standardized with mean = 0 and SD = 1.

^c^Adjusted for age (>50 years, yes/no) and BMI at baseline.

Sensitivity analyses did not substantively differ from our primary findings (Supplementary Tables S1–S5). After restricting the sample to diagnoses between 6 months and 4 years following study entry (Supplementary Table S1), the associations between iron levels and tumor size remained near null. The largest association was −0.055 (−0.105 to −0.004) between tumor size and serum ferritin, indicating a 0.055 cm decrease in the tumor size for a one unit SD increase in serum ferritin. Similarly, excluding people who reported taking iron supplements 4+ days/week did not alter the near null associations. In stratifying the primary analyses by tumor subtype (Supplementary Table S2), the associations shifted from inverse to positive for the HR^−^/HER2^−^ group. However, all associations remained near null, with wide 95% confidence intervals per one SD increase in the iron level exposure, but not exceeding a 0.05 cm change in tumor size. When evaluating median tumor size by a dichotomous threshold indicating extreme iron levels (Supplementary Table S3), differences in tumor size across the iron level groups did not generally exceed 0.3 cm. One exception was for premenopausal tumors with ferritin ≥300 mcg/dL. However, this group representing the extreme iron level was small (*n* = 16). In evaluating iron levels across breast cancer stage (Supplementary Table S4) and grade (Supplementary Table S5), we did not find any notable patterns deviating from our original findings.

## Discussion

We assessed associations between several serum iron measures at baseline and breast cancer tumor size and metastatic status in a large sample of women from a contemporary U.S. prospective cohort. All estimated associations were close to zero with no evidence to support our hypothesis of a positive association between body iron levels and tumor size.

We previously used the same sample to evaluate the association between serum iron levels and breast cancer incidence, (8) and did not observe strong evidence of an association between serum iron levels and breast cancer incidence. Our current findings regarding iron levels and tumor size outcomes also reflect near null associations. The included sensitivity analyses are consistent with these findings. One interesting *post hoc* finding after investigating associations between iron levels and breast cancer incidence suggests a potential protective association with lower iron levels. Specifically, breast cancer risk was lower for the lowest quartiles for serum iron and ferritin compared with the grouped top three quartiles. In our sensitivity analyses for extreme iron levels and tumor size (Supplementary Table S3), we did not find any evidence of a protective effect of very low iron levels, except that tumors were larger in premenopausal women with high ferritin. The consistency we found across different outcomes and a range of sensitivity analyses reinforces the lack of evidence to support higher serum iron levels being associated with adverse tumor-related outcomes in this sample.

One limitation of this study is the potential for variation over time—iron measures at baseline may not represent iron status when the tumor was developing. However, after examining associations for incident cancers more proximal to the blood draw through the sensitivity analyses, we did not find evidence to support positive associations between body iron levels and tumor size. Also, studies incorporating longitudinal iron level measures may have the potential to provide more insight into associations with tumor size. An advantage of this study was the amount of information collected from participants, which we could use in sensitivity analyses, including stratification by tumor subtype.

Few studies have examined these associations between body iron levels and tumor size outcomes, and replication of these results is necessary. Compared with findings from animal models, our findings suggest that serum iron levels in humans are not positively associated with tumor growth. Nevertheless, iron is highly regulated in the body with a complex feedback mechanism, and the human tumor microenvironment may operate independently of circulating serum iron levels.

## Supplementary Material

Supplemental Table 1 Supplemental Table 1: Associations between iron biomarkers, tumor size, and metastatic status (excluding diagnoses within 6 months of baseline and over 4 years after baseline and women who took iron supplements 4+ days/week at baseline)

Supplemental Table 2 Supplemental Table 2: Linear slope estimates for largest tumor sizea by iron measure and tumor subtype

Supplemental Table 3 Supplemental Table 3: Median largest tumor size by menopause status at baseline and iron status extreme thresholds

Supplemental Table 4 Supplemental Table 4: Comparison of baseline iron values by breast cancer stage

Supplemental Table 5 Supplemental Table 5: Comparison of baseline iron values by breast cancer grade

Supplemental Figure 1 Supplemental Figure 1: Flow diagram for the analytic sample of 2,494 women with a tumor size measure from the total of 3,007 diagnosed with invasive or ductal carcinoma in situ (DCIS) breast cancer.

## References

[1] Torti SV , Torti FM. Iron: the cancer connection. Mol Aspects Med 2020;75:100860. 32340745 10.1016/j.mam.2020.100860 PMC9107937

[2] Pfeifhofer-Obermair C , Tymoszuk P, Petzer V, Weiss G, Nairz M. Iron in the tumor microenvironment-connecting the dots. Front Oncol 2018;8:549. 30534534 10.3389/fonc.2018.00549 PMC6275298

[3] Hann HWL , Stahlhut MW, Menduke H. Iron enhances tumor growth. Observation on spontaneous mammary tumors in mice. Cancer 1991;68:2407–10. 1657354 10.1002/1097-0142(19911201)68:11<2407::aid-cncr2820681113>3.0.co;2-n

[4] Le NTV , Richardson DR. Iron chelators with high antiproliferative activity up-regulate the expression of a growth inhibitory and metastasis suppressor gene: a link between iron metabolism and proliferation. Blood 2004;104:2967–75. 15251988 10.1182/blood-2004-05-1866

[5] Basuli D , Tesfay L, Deng Z, Paul B, Yamamoto Y, Ning G, . Iron addiction: a novel therapeutic target in ovarian cancer. Oncogene. 2017;36:4089–99. 28319068 10.1038/onc.2017.11 PMC5540148

[6] Chang VC , Cotterchio M, Khoo E. Iron intake, body iron status, and risk of breast cancer: a systematic review and meta-analysis. BMC Cancer 2019;19:543. 31170936 10.1186/s12885-019-5642-0 PMC6555759

[7] Sandler DP , Hodgson ME, Deming-Halverson SL, Juras PS, D'Aloisio AA, Suarez LM, . The Sister Study cohort: baseline methods and participant characteristics. Environ Health Perspect 2017;125:127003. 29373861 10.1289/EHP1923 PMC5963586

[8] Von Holle A , O'Brien KM, Sandler DP, Janicek R, Weinberg CR. Association between serum iron biomarkers and breast cancer. Cancer Epidemiol Biomarkers Prev 2021;30:422–5. 33293341 10.1158/1055-9965.EPI-20-0715 PMC7867615

