# Supplementary material for: Associations Between Serum Iron Biomarkers and Breast Cancer Tumor Size
Source: Cancer Res Commun. 2024 Jan 23;4(1):182–5. doi: 10.1158/2767-9764.CRC-23-0205 (PMC10804913; doi:10.1158/2767-9764.CRC-23-0205)
Supplement: Supplemental Table 1 — Associations between iron biomarkers, tumor size, and metastatic status (excluding diagnoses within 6 months of baseline and over 4 years after baseline and women who took iron supplements 4+ days/week at baseline) [file crc-23-0205-s01.pdf]

Supplemental Table 1: Associations between iron biomarkers, tumor size, and metastatic status (excluding diagnoses within 6 months of baseline and over 4 years after baseline and women who took iron supplements 4+ days/week at baseline)

		Largest tumor size[a]		Metastatic outcome (yes vs no)	
Exposure[b]	Spearman's Rank Correlation	Unadjusted β	Adjusted β^c	Odds ratio (95% CI)	Odds Ratio (95% CI)[c]
**Including only diagnoses >= 6 months and < 4 years after baseline iron measurement (n = 975)**					
Iron (mcg/dL)	-0.024	-0.020 (-0.070, 0.030)	-0.017 (-0.068, 0.034)	1.162 (0.579, 2.331)	1.132 (0.560, 2.287)
Ferritin (mcg/dL)	-0.059	-0.057 (-0.107, -0.007)	-0.055 (-0.105, -0.004)	1.082 (0.604, 1.939)	1.089 (0.645, 1.836)
Transferrin saturation (%)	-0.018	-0.006 (-0.057, 0.046)	-0.002 (-0.055, 0.050)	1.009 (0.481, 2.118)	0.984 (0.465, 2.080)
**Excluding women who took iron supplements 4+ days/week (n = 2,271)**					
Iron (mcg/dL)	-0.013	-0.012 (-0.044, 0.021)	-0.013 (-0.046, 0.020)	1.275 (0.749, 2.169)	1.319 (0.748, 2.326)
Ferritin (mcg/dL)	-0.028	-0.039 (-0.072, -0.007)	-0.032 (-0.065, 0.001)	1.128 (0.768, 1.657)	1.129 (0.758, 1.679)
Transferrin saturation (%)	-0.016	-0.009 (-0.042, 0.024)	-0.010 (-0.044, 0.023)	1.073 (0.603, 1.909)	1.088 (0.598, 1.978)

[a] Natural log-transformed
[b] Standardized with mean = 0 and standard deviation = 1
[c] Adjusted for age (>50 years, yes/no) and BMI at baseline
