# Supplementary material for: Associations Between Serum Iron Biomarkers and Breast Cancer Tumor Size
Source: Cancer Res Commun. 2024 Jan 23;4(1):182–5. doi: 10.1158/2767-9764.CRC-23-0205 (PMC10804913; doi:10.1158/2767-9764.CRC-23-0205)
Supplement: Supplemental Table 2 — Linear slope estimates for largest tumor sizea by iron measure and tumor subtype [file crc-23-0205-s02.pdf]

Supplemental Table 2: Linear slope estimates for largest tumor size[a] by iron measure and tumor subtype

		Largest tumor size[a]	
Tumor subtype and exposure[b]	Spearman's Rank Correlation	Unadjusted β	Adjusted β^c
**HR+/HER2- (n = 1,617)**			
Iron (mcg/dL)	-0.030	-0.025 (-0.061, 0.010)	-0.027 (-0.064, 0.009)
Ferritin (mcg/dL)	-0.054	-0.038 (-0.073, -0.002)	-0.030 (-0.067, 0.006)
Transferrin saturation (%)	-0.031	-0.025 (-0.061, 0.012)	-0.027 (-0.064, 0.010)
**HR+/HER2+ or HR-/HER2+ (n = 243)**			
Iron (mcg/dL)	-0.117	-0.040 (-0.134, 0.054)	-0.035 (-0.131, 0.060)
Ferritin (mcg/dL)	-0.026	-0.028 (-0.122, 0.066)	-0.024 (-0.120, 0.072)
Transferrin saturation (%)	-0.113	-0.050 (-0.144, 0.044)	-0.048 (-0.144, 0.049)
**HR-/HER2- (n = 201)**			
Iron (mcg/dL)	0.051	0.037 (-0.068, 0.142)	0.040 (-0.069, 0.149)
Ferritin (mcg/dL)	0.019	0.035 (-0.070, 0.140)	0.037 (-0.071, 0.144)
Transferrin saturation (%)	0.024	0.035 (-0.075, 0.144)	0.042 (-0.073, 0.158)

[a] Natural log-transformed
[b] Standardized with mean = 0 and standard deviation = 1
[c] Adjusted for age (>50 years, yes/no) and BMI at baseline
