# Supplementary material for: Associations Between Serum Iron Biomarkers and Breast Cancer Tumor Size
Source: Cancer Res Commun. 2024 Jan 23;4(1):182–5. doi: 10.1158/2767-9764.CRC-23-0205 (PMC10804913; doi:10.1158/2767-9764.CRC-23-0205)
Supplement: Supplemental Table 3 — Median largest tumor size by menopause status at baseline and iron status extreme thresholds [file crc-23-0205-s03.pdf]

Supplemental Table 3: Median largest tumor size by menopause status at baseline and iron status extreme thresholds

High/low indictor	Iron measure	Index group (by dichotomized iron threshold)	Index group		Referent group	
			Tumor size, median (IQR)	n	Tumor size, median (IQR)	n
**Premenopausal**						
High	Ferritin	>=300	0.6 (0.4 - 1.3)	16	1.5 (0.9 - 2.3)	731
	Transferrin Saturation	>=45	1.4 (1 - 2)	127	1.5 (0.9 - 2.3)	665
	Transferrin Saturation	>=50	1.5 (0.9 - 2.2)	90	1.5 (0.9 - 2.2)	699
	Transferrin Saturation	>=55	1.4 (0.9 - 2.4)	72	1.5 (0.9 - 2.2)	713
	Transferrin Saturation	>=60	1.4 (0.8 - 2.2)	60	1.5 (0.9 - 2.3)	723
Low	Ferritin	<=12	1.5 (0.9 - 2.3)	680	1.5 (1 - 2.1)	77
	Ferritin	<=25	1.5 (0.9 - 2.2)	532	1.5 (1 - 2.4)	228
	Ferritin	<=45	1.5 (0.9 - 2.3)	349	1.5 (0.9 - 2.2)	406
	Transferrin Saturation	<=20	1.5 (0.9 - 2.2)	598	1.5 (0.9 - 2.5)	201
**Postmenopausal**						
High	Ferritin	>=300	1.4 (0.8 - 1.8)	38	1.3 (0.8 - 2)	1720
	Transferrin Saturation	>=45	1.3 (0.8 - 2.2)	241	1.3 (0.8 - 2)	1637
	Transferrin Saturation	>=50	1.2 (0.7 - 1.9)	167	1.3 (0.8 - 2)	1699
	Transferrin Saturation	>=55	1.1 (0.8 - 1.9)	131	1.3 (0.8 - 2)	1731
	Transferrin Saturation	>=60	1 (0.6 - 1.6)	116	1.3 (0.8 - 2)	1743
Low	Ferritin	<=12	1.3 (0.8 - 2)	1723	1 (0.6 - 1.6)	42
	Ferritin	<=25	1.3 (0.8 - 2)	1609	1.2 (0.8 - 1.9)	159
	Ferritin	<=45	1.3 (0.8 - 2)	1373	1.3 (0.8 - 2)	396
	Transferrin Saturation	<=20	1.3 (0.8 - 2)	1553	1.3 (0.8 - 2.1)	355
