# Supplementary material for: Associations Between Serum Iron Biomarkers and Breast Cancer Tumor Size
Source: Cancer Res Commun. 2024 Jan 23;4(1):182–5. doi: 10.1158/2767-9764.CRC-23-0205 (PMC10804913; doi:10.1158/2767-9764.CRC-23-0205)
Supplement: Supplemental Table 4 — Comparison of baseline iron values by breast cancer stage [file crc-23-0205-s04.pdf]

Supplemental Table 4: Comparison of baseline iron values by breast cancer stage

**Characteristic**	**0**, N = 625	**I**, N = 1,444	**II**, N = 567	**III**, N = 108	**IV**, N = 21	**p-value**
Ferritin, ug/dL	66 (37, 111)	72 (39, 120)	66 (35, 115)	61 (30, 107)	78 (64, 133)	0.076
Iron, ug/dL	93 (74, 114)	94 (76, 116)	90 (73, 114)	90 (74, 113)	98 (87, 119)	0.14
Transferrin Saturation, ug/dL	29 (23, 36)	29 (23, 36)	28 (22, 35)	28 (22, 36)	30 (24, 36)	0.22

[1] Median (IQR)

[2] Kruskal-Wallis rank sum test
