# Supplementary material for: Associations Between Serum Iron Biomarkers and Breast Cancer Tumor Size
Source: Cancer Res Commun. 2024 Jan 23;4(1):182–5. doi: 10.1158/2767-9764.CRC-23-0205 (PMC10804913; doi:10.1158/2767-9764.CRC-23-0205)
Supplement: Supplemental Table 5 — Comparison of baseline iron values by breast cancer grade [file crc-23-0205-s05.pdf]

Supplemental Table 5: Comparison of baseline iron values by breast cancer grade

**Characteristic**	**1**, N = 566	**2**, N = 933	**3**, N = 523	**p-value**
Ferritin, ug/dL	72 (39, 120)	69 (38, 118)	70 (34, 116)	0.83
Iron, ug/dL	94 (76, 116)	94 (76, 117)	92 (74, 115)	0.53
Transferrin Saturation, ug/dL	28 (23, 36)	29 (22, 36)	29 (22, 36)	0.87

[1] Median (IQR)

[2] Kruskal-Wallis rank sum test
