# Supplementary material for: Associations Between Serum Iron Biomarkers and Breast Cancer Tumor Size
Source: Cancer Res Commun. 2024 Jan 23;4(1):182–5. doi: 10.1158/2767-9764.CRC-23-0205 (PMC10804913; doi:10.1158/2767-9764.CRC-23-0205)
Supplement: Supplemental Figure 1 — Flow diagram for the analytic sample of 2,494 women with a tumor size measure from the total of 3,007 diagnosed with invasive or ductal carcinoma in situ (DCIS) breast cancer. [file crc-23-0205-s06.pdf]

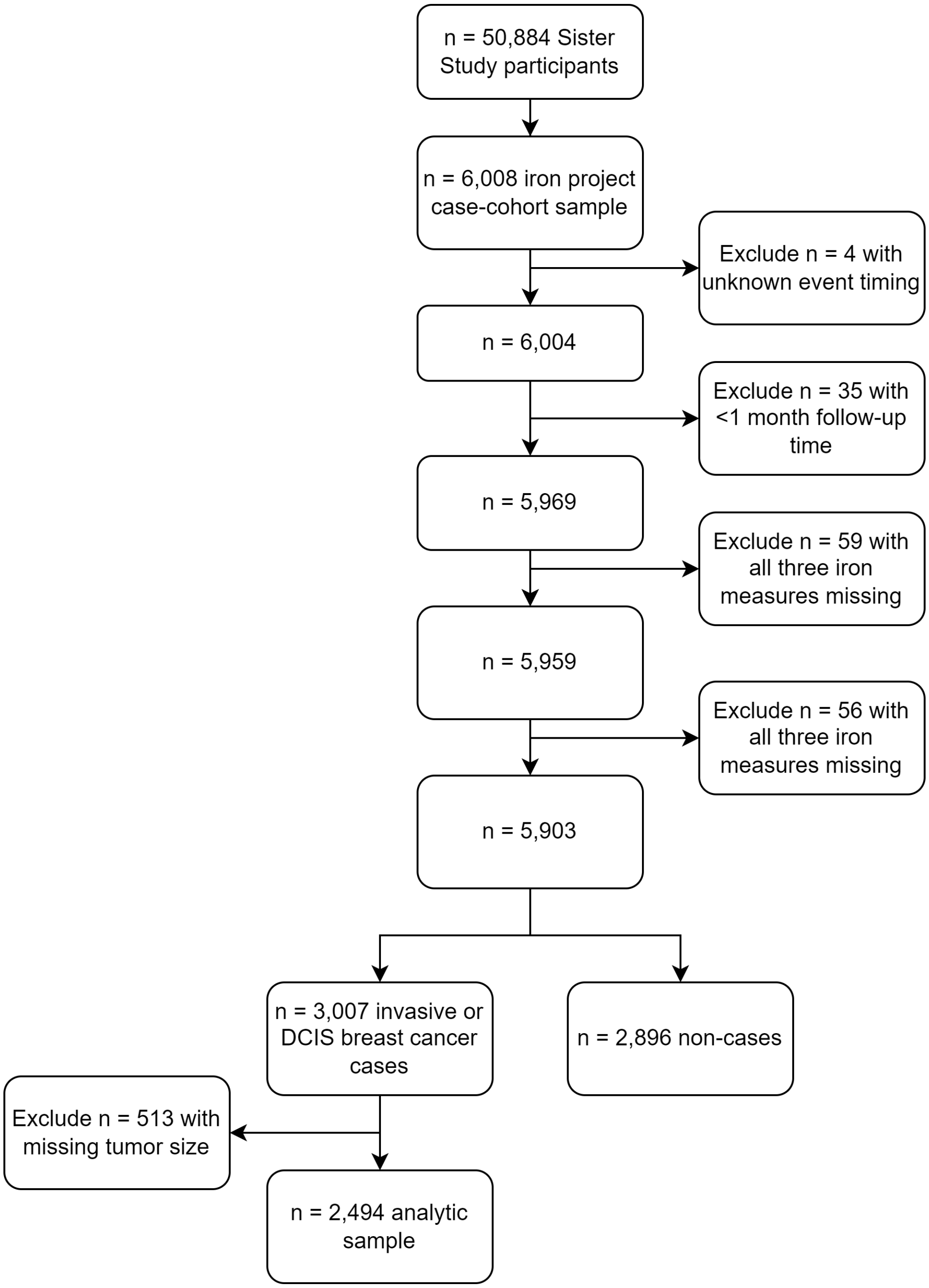

Supplemental Figure 1: Flow diagram for the analytic sample of 2,494 women with a tumor size measure from the total of 3,007 diagnosed with invasive or ductal carcinoma in situ (DCIS) breast cancer.
